# Solar Air-to-Fuel Technologies for a Circular Carbon Economy

**DOI:** 10.1021/jacs.5c21750

**Published:** 2026-03-04

**Authors:** Sayan Kar, Erwin Reisner

**Affiliations:** † Department of Sustainable Energy Engineering, 30077 Indian Institute of Technology Kanpur, Kanpur, Uttar Pradesh 208016, India; ‡ Yusuf Hamied Department of Chemistry, 2152 University of Cambridge, Cambridge CB2 1EW, U.K.

## Abstract

The CO_2_ present in our atmosphere is a universally available and abundant carbon feedstock, but it exists only in dilute concentrations (currently around 427 ppm). Clean technologies capable of capturing atmospheric CO_2_ using direct air capture and directly converting it into synthetic fuels and chemicals using solar energy could pave the way for a circular chemical industry. Challenges in developing such a solar-powered air-to-fuel technology that mimics photosynthesis include the use of the low CO_2_ concentration in air, the thermodynamic stability of CO_2_ (and its capture products), the high O_2_ content (21%) in air (which often interferes with catalytic CO_2_ valorization), and the presence of variable moisture levels. This perspective explores different concepts and emerging technologies for the solar-powered conversion of atmospheric CO_2_ into fuels and chemicals, examines their scientific principles, considers their scalability, and offers recommendations for future research to support the development of a net-zero circular carbon economy.

## Graphical abstract

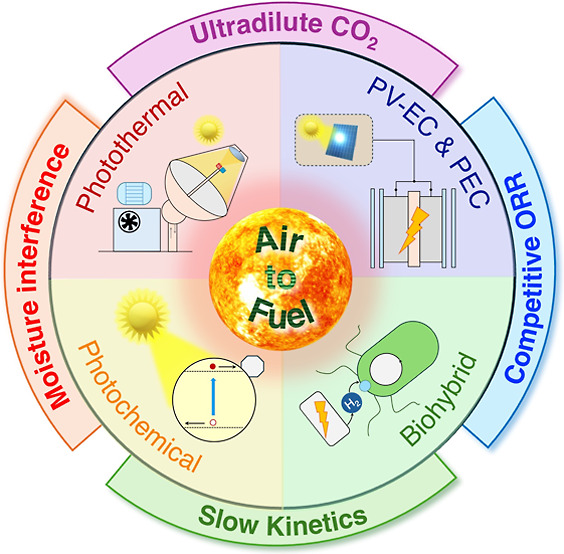

## 1 Introduction

Earth’s natural carbon cycle plays a crucial role in sustaining life on our planet. Until the industrial revolution, the atmospheric carbon dioxide (CO_2_) levels had largely remained stable (at below 300 ppm) for several thousand years, with forests, soil, and oceans sequestering nearly equal amounts of CO_2_ to those being released by the natural processes (Figure 1).^1,2^ Since the industrialization of our planet over the past 250 years, vast quantities of additional CO_2_ have been and continue to be released into the atmosphere by using the planet’s fossil fuel reserves to meet the energy demands of our civilization.^3^ This additional anthropogenic CO_2_ emission (currently at 30–40 billion tons per year) is causing a carbon cycle imbalance. Consequently, atmospheric CO_2_ levels continue to rise, and while its effects in the form of global warming, climate change, ecological destabilization, ocean acidification, and others have long been predicted, they are now beginning to have a profound impact on our planet.^4–7^

**Figure 1:**
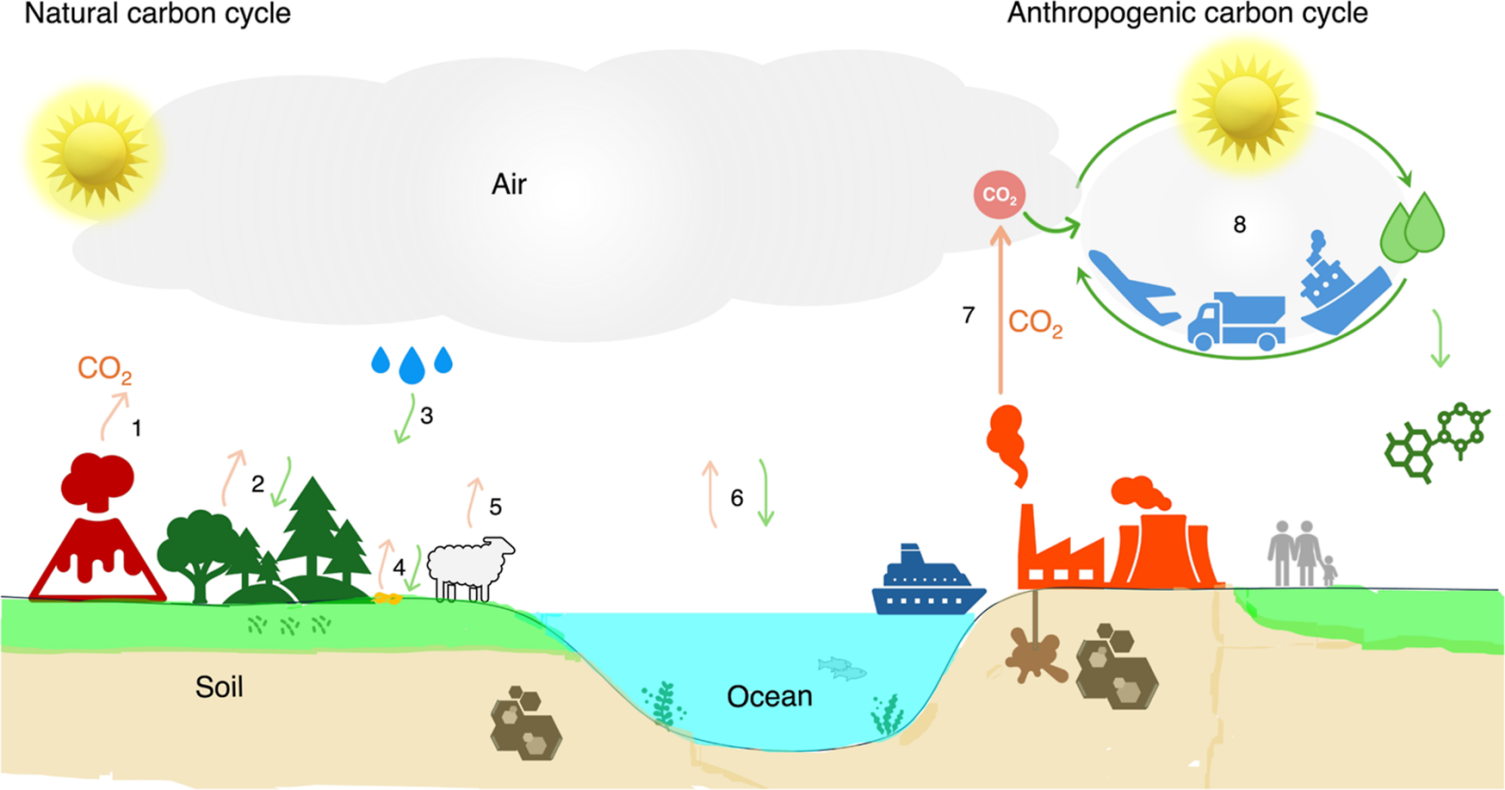
The net zero carbon cycle of the future. The natural circular carbon cycle is illustrated on the left, showing different carbon fluxes to and from air (nonexhaustive), including those from natural activities (1), forests (2), rainfall (3), topsoil (4), animals (5), and oceans (6). All natural fluxes combine to a total CO_2_ emission of around zero. Flux 7 represents the anthropogenic CO_2_ emission that currently enters the air without removal. The top-right corner illustrates the envisioned anthropogenic circular carbon cycle (8) through solar CO_2_ recycling into fuels from air for a circular carbon economy.

To address this, significant attention is being devoted to reducing net CO_2_ emissions on a global scale.^8^ In devising future solutions, it is essential to recognize our reliance on carbon; as all life is composed of carbon, our economy runs on carbon, and our everyday materials largely depend on carbon. Anthropogenic carbon circularity can be achieved through a nature-inspired approach, where we develop efficient ways to capture and manipulate CO_2_ from the air for everyday fuel and chemical synthesis.^9,10^ This air-to-fuel approach will not only enable us to recycle or reduce atmospheric CO_2_ but also minimize our reliance on fossilized carbon.^11–14^ Aerobic utilization of CO_2_ to produce fuels enables an anthropogenic net-zero carbon cycle, where CO_2_ is reemitted into the atmosphere when the recycled fuel is used, closing the loop. Alternatively, aerobic CO_2_ fixation into long-lasting chemicals, such as polymers, yields a carbon-negative cycle but is constrained by the limited amount of polymer required in human society.^15,16^ Another related carbon-negative approach is the capture and subsequent storage of atmospheric CO_2_ in geological formations, as in direct air carbon capture and storage (DACCS); however, the safety, prudence, and economic viability of storing gigatons of pressurized CO_2_ underground remain the subject of considerable debate.^17–20^

Among the ways to harness and chemically valorize CO_2_ directly from air, the direct solar-powered route is ambitious but arguably the most promising long-term solution due to the abundance and the low cost of solar energy.^21,22^ As in natural photosynthesis, solar-powered air-to-fuel technologies aim to produce energy-rich carbon-based fuels directly from atmospheric CO_2_, utilizing sunlight as the energy source (Figure 1).^23–25^ This approach offers several benefits: (i) it utilizes atmospheric CO_2_ directly instead of relying on localized concentrated streams, (ii) it employs sunlight as a clean, emission-free, and abundant energy source,^26^ and (iii) it creates value by synthesizing targeted fuels and chemicals tailored to our needs instead of solely storing CO_2_ in the ground,^27^ as envisaged by conventional DACCS.^19,20^ Considerations regarding the required efficiency and scalability make solar air-to-fuel conversion a formidable challenge. While nature has been carrying out this process for millions of years using photosynthetic organisms,^28^ artificial systems for the process have only recenly been explored and are at an early formative stage. These recent developments will be covered in this perspective.

## 2 Challenges

### 2.1 Ultradilute CO_2_ Concentration

One key challenge in working with atmospheric CO_2_ is its low concentration in air, currently around 427 ppm (Figure 2a). Thermodynamically, it requires approximately 19.2 kJ mol_CO2_^–1^, corresponding to 0.44 GJ ton_CO2_^–1^, to concentrate ambient CO_2_ from atmospheric levels to pure CO_2_ at 1 bar (at temperature 25 °C) , which most solar utilization technologies would use as feedstock to avoid slow reaction kinetics arising from otherwise low substrate availability and slow mass transport (Figure 2b). Taking into account suitable kinetics, heat losses, efficiency losses, and the energy needed for air intake, current direct air capture (DAC) systems consume in reality between 5 and 9 GJ ton_CO2_^–1^.^29^ The majority of DAC technologies operate through chemical CO_2_ capture, where CO_2_ is first captured (absorbed or adsorbed) chemically and then extracted as a concentrated stream using a temperature or pressure swing.^30^ The low CO_2_ concentration in air necessitates capturing agents with high CO_2_ fixation rates (lowest activation energy) for suitable kinetics that are often associated with greater thermodynamic stability of the captured CO_2_ (Figure 2b), making subsequent release energy-intensive. Typical DAC materials utilize ethanolamines, specialized amines, or hydroxide-based agents as the active components,^30,31^ with extensive material development ongoing to create improved energy-efficient DAC chemical systems with faster reaction kinetics and lower thermodynamic energy penalty.^32–35^

**Figure 2:**
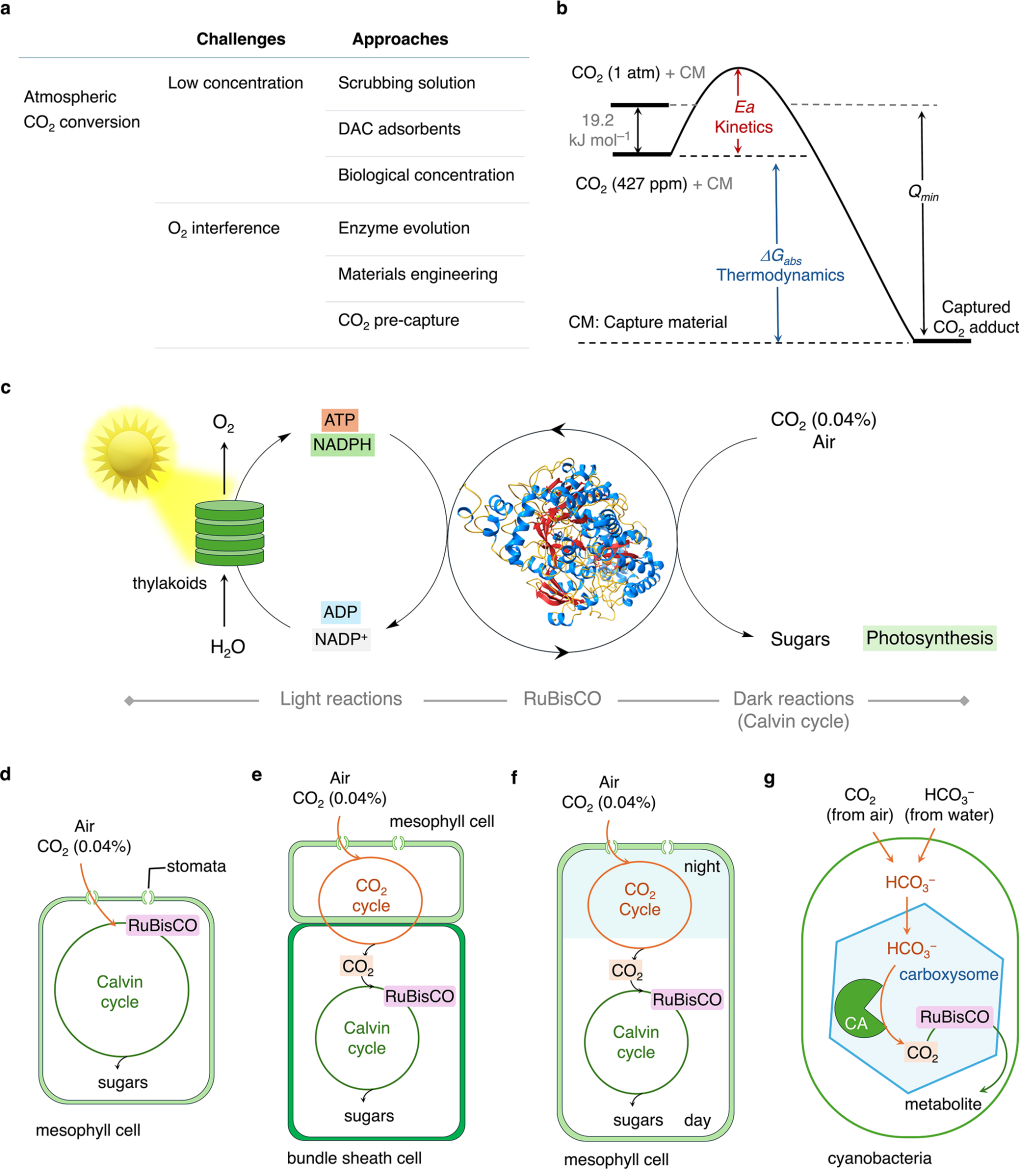
Challenges in CO_2_ reduction from air and nature’s solutions. (a) The challenges and various approaches, (b) the thermodynamic and kinetic considerations (*E*_a_, Δ*G*_abs_, and *Q*_min_ refer to the activation energy, free energy change during CO_2_ absorption, and minimum work for release to 1 atm CO_2_, respectively), and (c) natural atmospheric CO_2_ fixation during photosynthesis enabled by the RuBisCO enzyme. The RuBisCO structure is taken from the National Institutes of Health database and is available in the public domain.^56^ (d–g) Various CO_2_-relaying and concentrating mechanisms observed in nature to deliver atmospheric CO_2_ to RuBisCO, including in C3 plants (d), C4 plants (e), CAM plants (f), and cyanobacteria (g) (CA denotes carbonic anhydrase).

Beyond thermal processes, innovative DAC methods powered by alternative energy sources are also being explored. These include hybrid electrochemical DAC systems with electrochemically active redox species for reversible CO_2_ capture,^36–38^ electrochemically mediated amine regeneration techniques via the competitive binding of a redox-active metal ion with amines,^39–42^ moisture swing DAC adsorbents for regeneration through a humidity alteration in the feed gas,^43–45^ electrochemically or photochemically triggered pH swings for reversible CO_2_ capture and release,^46–49^ and membrane-based CO_2_ separation from air.^50,51^ Comprehensive analyses of these systems have recently been undertaken.^52–54^ These emerging technologies are in early stages and require further development before they can be used in DAC facilities.

CO_2_ from air can also be used directly for conversion without prior concentration to avoid the DAC energy penalty, but the low CO_2_ concentration makes the reaction kinetics in such systems sluggish.^55^ This is often exacerbated by interference from other high-concentration reactive species in the air such as oxygen and moisture.

### 2.2 High Oxygen (O_2_) Content

The O_2_ concentration in the atmosphere is 21%, approximately 500 times that of CO_2_. Unlike the most abundant and mostly inert gas in the atmosphere, nitrogen (N_2_; 79%), O_2_ is a reactive diradical that can interfere with the CO_2_ conversion process. The facile nature of the oxygen reduction reaction is reflected in its positive and thus favorable reduction potential (to OH^–^, H_2_O_2_,_,_ or H_2_O), compared to CO_2_ reduction reactions.^57^ Nature has addressed this problem in photosynthesis by developing the ribulose-1,5-bisphosphate carboxylase/oxygenase (RuBisCO) enzyme, which is several orders (∼10^3^) more active in reducing CO_2_ than O_2_ (Figure 2c).^58,59^ Several factors, including high active-site affinity, enhanced local solubility, and stabilizing side-chain interactions, contribute to RuBisCO’s preference for reducing CO_2_ over O_2_, which nature has refined over millions of years.^60,61^ Even so, for plants lacking a local CO_2_ concentration mechanism where CO_2_ is uptaken through the leaf stomate and directly relayed to RuBisCO in mesophyll cells (C3 plants), RuBisCO is known to reduce, on average, one O_2_ molecule (photorespiration, hence the term oxygenase in its name) for every three molecules of CO_2_ (photosynthesis), reducing the overall photosynthesis efficiency by approximately 25% (Figure 2d).^62,63^

### 2.3 Nature’s CO_2_ Concentrating Mechanisms

Selectivity for CO_2_ reduction is enhanced in nature through a preconcentration mechanism in C4 plants, where atmospheric CO_2_ is initially fixed as part of a four-carbon compound within mesophyll cells and then released in the bundle sheath cells at higher concentrations around RuBisCO, where the Calvin cycle (dark reactions) takes place (Figure 2e).^64,65^ Alternate ways to improve CO_2_ reduction selectivity, as seen in nature, include temporal separation of CO_2_ uptake and reduction, as in CAM (Crassulacean Acid Metabolism) plants, where CO_2_ is stored overnight and released for reduction during the day (Figure 2f),^66,67^ or the local release of concentrated CO_2_ via pH swing within microcompartments called carboxysomes found in cyanobacteria. Carboxysomes contain the enzymes carbonic anhydrase and RuBisCO close to each other, where carbonic anhydrase facilitates the local concentrated CO_2_ release, and RuBisCO fixes the released CO_2_ in situ (Figure 2g).^68,69^ Devising synthetic methods to achieve a similar competitive advantage in CO_2_ reduction in the presence of 21% O_2_ has proved challenging, with prominent approaches being surface hydrophobic treatment, catalytic molecular tuning, and introduction of CO_2_ transport relays to active sites, among others.^57,70–72^

### 2.4 Temporal Decoupling of Capture and Reduction

A nature-inspired strategy to avoid O_2_ interference has been the temporal decoupling of the CO_2_ capture and conversion processes into separate steps, as in CAM plants, rather than simultaneous capture and conversion (Figure 3).^73,74^ The common chemicals used in CO_2_ capture (including DAC), such as amines, ethanolamines, and hydroxides, are unreactive toward nonelectrophilic O_2_. Thus, CO_2_ can be selectively separated and captured in the solution by these chemicals while allowing other gases, such as N_2_ and O_2_, to pass through. The CO_2_-loaded solution can then release pure CO_2_ gas using solar power. Such a decoupled system can operate throughout the full diurnal cycle, using night-time to concentrate aerobic CO_2_ in solution and day-time for the release and conversion of pure CO_2_.^75^

**Figure 3:**
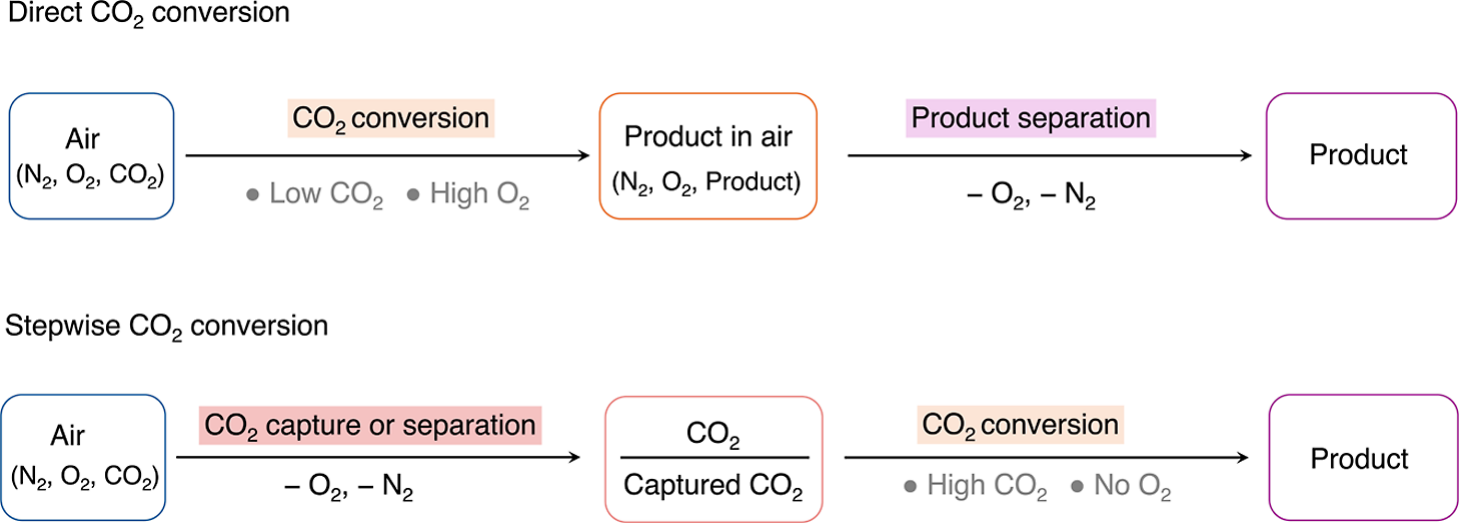
Direct and stepwise air capture and conversion. The direct route consists of direct CO_2_ conversion at low CO_2_ concentrations in the presence of O_2_. In the stepwise approach, CO_2_ is first captured or separated from N_2_ and O_2_, followed by its conversion.

### 2.5 Presence of Moisture and Other Impurities

Complications can arise from the presence of moisture in air, which fluctuates throughout the diurnal cycle. For physisorbing DAC materials based on porous, ordered structures (zeolites, metal–organic frameworks), humidity can substantially reduce their CO_2_ capture performance due to competitive binding within the pores.^76^ Conversely, chemisorbing DAC materials (especially amine-based ones) exhibit improved CO_2_ capture rates and capacities in humid conditions, but coadsorption of water increases the energy intensity of desorption while decreasing the purity and recovery rate of CO_2_.^77–79^ The presence of moisture in the recovered CO_2_ can further interfere with its downstream conversion, often necessitating additional CO_2_ drying or moisture-stabilizing steps. In addition, in many electro- and photo-catalytic CO_2_ utilization processes, some moisture is required to supply the protons needed for the CO_2_-reduction chemistry to proceed.

Besides O_2_ and moisture, other impurities present in air, such as NO_*x*_ and SO_*x*_, can potentially interfere with DAC or conversion, but their aerobic concentration is typically low enough (parts per billion levels) to avoid any prominent effect. This contrasts with CO_2_ capture and utilization from dilute industry sources such as postcombustion flue gases where elevated SO_*x*_ and NO_*x*_ levels are commonly observed (100–1000 ppm).^72,80^

## 3 Status

Several innovations have been reported recently for the direct synthesis of fuels and chemicals from atmospheric CO_2_, with sunlight serving as the primary energy source. These technologies utilize various approaches, including physical, chemical, and biological CO_2_ fixation, combined with thermochemical, electrochemical, photochemical, and biochemical conversion pathways to achieve solar air-to-fuel synthesis, as detailed below.

### 3.1 The Solar–Thermochemical Pathway

#### 3.1.1 Thermochemical Looping

CO_2_ can be split into CO and O_2_ at high temperatures via a thermochemical cycle using oxygen-transport materials, such as ceria (CeO_2_) (Figure 4a).^81,82^ CeO_2_, when treated at high temperatures (above 1000 °C), releases O_2_ from its structure to form reduced oxygen-vacant ceria (CeO_2−δ_, δ up to 0.04). Reduced CeO_2_, when exposed to CO_2_ or H_2_O at a lower temperature (<1000 °C), extracts oxygen from these feedstocks to fill its oxygen vacancy while producing carbon monoxide (CO) and hydrogen (H_2_), respectively. Besides CeO_2_, other oxygen-transport materials can also be used to split H_2_O and CO_2_ at high temperatures, including zinc oxide, metal-doped iron oxides, iron–aluminum oxides, and perovskite materials.^83–87^ The material must maintain a stable lattice structure at the required high temperatures (∼1500 °C) under repeated heating and cooling cycles for a prolonged period to be effective for continuous operation. The process can be solar-driven when concentrated sunlight is used to achieve high temperatures. When sunlight is used directly to heat the transport material, it is termed direct thermochemical splitting. Conversely, if concentrated solar heat is transferred via a heat-transfer liquid, it is termed an indirect process.^88^

**Figure 4:**
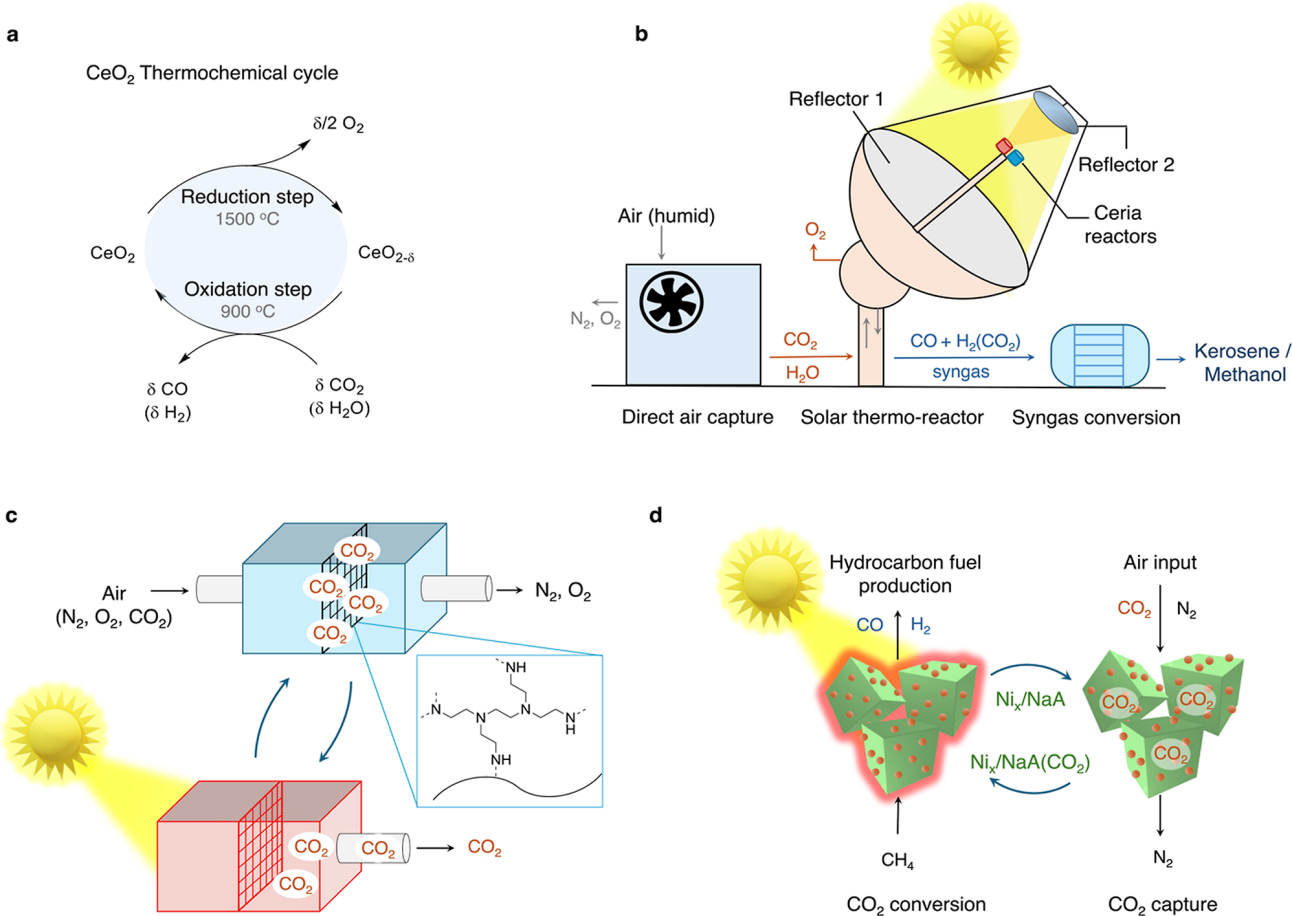
Solar–thermal air-to-fuel systems. (a) CeO_2_-based thermochemical cycle, (b) the solar–thermal air-to-fuel technology with three distinct steps^89^ (the red and blue ceria reactors represent the “hot” (1500 °C) and “cold” (900 °C) chambers, respectively), (c) the direct air capture system using immobilized amines,^90^ and (d) solar–thermal air-to-fuel technology using dual functional materials for CO_2_ capture and conversion.^97^

Most high-temperature CO_2_ splitting reports based on thermochemical looping using oxygen-transport materials have focused on using prepurified concentrated CO_2_. Recently, Steinfeld and co-workers have demonstrated its integration with direct air CO_2_ capture to produce drop-in fuels from sunlight and air (Figure 4b).^89^ In the first step, CO_2_ and moisture are captured from the air using well-established solid amine-based adsorbents developed by the same group in the early 2010s (now pursued through the spin-off company Climeworks).^90,91^ These adsorbents release captured CO_2_ at moderate temperatures (80–100 °C) or under reduced pressures (Figure 4c). In the developed technology, air-captured CO_2_ is released under mild heat (100 °C, obtained from waste heat of the solar refinery, vide infra) at reduced pressure (<0.1 bar) and fed to a solar refinery at ambient pressure, where the second step, conversion to syngas (a mixture of CO and H_2_, which is an industrial fuel and chemical precursor), takes place.

The solar refinery employs porous CeO_2_ blocks, which are initially heated to 1500 °C using highly concentrated solar light (intensities of up to 3000–5000 kW m^–2^, equivalent to 3000–5000 suns) via a parabolic dish collector (Figure 4b).^92^ This step, also known as the reduction or oxygen-release step, is performed under reduced pressure (0.1mbar) to maximize oxygen vacancies in reduced ceria. In the subsequent oxidation or oxygen-uptake step, concentrated sunlight is moved away from the block, allowing it to cool down. Air-captured CO_2_ and H_2_O are then introduced at ambient pressure, around 900 °C, to complete the thermochemical cycle by restoring the oxygen atoms to ceria while producing syngas (Figure 4a). The porosity of the ceria block plays a crucial role and is tuned to balance two opposing factors: an increase in solar absorption with larger pore sizes, and an increase in reaction rate with smaller pore sizes due to an increase in the surface area of the block.^88,93^ The regenerated CeO_2_ is reused in the next thermochemical cycle.

This process can produce approximately 4 L of syngas per kilogram of CeO_2_ per cycle, demonstrated for a few hundred cycles, with a recorded solar-to-syngas efficiency of around 3–5%.^89^ The produced syngas can be utilized to produce liquid fuels in a downstream gas-to-liquid (GTL) unit, such as to methanol (marine fuel) using an industrially established Cu/ZnO/Al_2_O_3_ syngas-to-methanol catalyst or to kerosene (aviation fuel) via Fischer–Tropsch synthesis.^89^ The GTL conversions are typically conducted at around 200–300 °C by using waste heat from the solar refinery, making the process entirely solar-driven. The overall system efficiency of this air-to-fuel process was estimated to be around 1%. It has been scaled to 50 kW (∼50 m^2^ of solar irradiance) at IMDEA Energy in Spain for CO_2_-to-fuel production, albeit without the DAC component, and material stability over long reaction times poses a challenge.^94^ Other challenges include high capital expenditure requirements (for heliostat field), need for large amounts of porous CeO_2_ (critical mineral) and quartz, high radiative heat loss during operation, nonstoichiometric oxygen transfer, and material sintering at elevated temperatures, which reduce efficiency and longevity.^95,96^

#### 3.1.2 Solar Thermal Catalysis Using Dual Sorbent Materials

Besides thermochemical looping, dual-functional catalytic sorbents can also be used for DAC and its subsequent conversion to synthesis gas under concentrated sunlight (Figure 4d), as reported recently by Liu and co-workers.^97^ Unlike the thermochemical looping systems, where CO_2_ capture and utilization occur at separate locations with different materials, here the sorbent plays roles in both CO_2_ capture and conversion. The adsorbent consists of a commercial zeolite, NaA, which aids in CO_2_ capture, and is impregnated with nickel, which facilitates CO_2_ conversion. In a typical reaction setup, a CO_2_-containing gas (including simulated air) is first passed through the adsorbent at ambient temperature for preloading of CO_2_ (up to around 1 mmol per gram of the adsorbent). The adsorbent is then exposed to concentrated sunlight (around 410 sun intensity, 410.2 kW m^–2^) under CH_4_ flow, resulting in adsorbed CO_2_ release and its conversion to synthesis gas via dry methane reforming process with reaction temperatures reaching 700 °C under concentrated light illumination. CO_2_ conversion of up to 95% is reported under these conditions. However, using the same material as both the capture sorbent and the conversion catalyst limits the scale of the single-cycle reaction due to limited CO_2_ adsorption. Continuous switching between capture and conversion required for continuous operation can also be challenging.

### 3.2 The Photovoltaic-Driven Electrochemical Pathway

CO_2_ can also be recycled into fuels and chemicals using electrical energy, with or without the aid of high temperatures.^98,99^ High-temperature (typically operating above 500 °C) solid oxide electrolysis cells (SOECs) have been intensively investigated for CO_2_-to-fuel conversion and have been reviewed recently.^100–102^ The integration of SOECs with DAC systems has recently been envisioned.^103^ This process can be solar-driven if the required electricity and heat are generated with photovoltaic panels and concentrated solar thermal power, respectively. Electrochemical systems can also reduce CO_2_ without high temperatures and, consequently, without the thermodynamic Carnot losses. The reduction of pure CO_2_ using near-ambient-temperature electrolyzers has advanced rapidly in recent years, with current densities reaching A cm^–2^ levels.^104–106^ However, the electrochemical conversion of CO_2_ directly from air remains challenging due to low CO_2_ and high O_2_ concentrations (Figure 5a).

**Figure 5:**
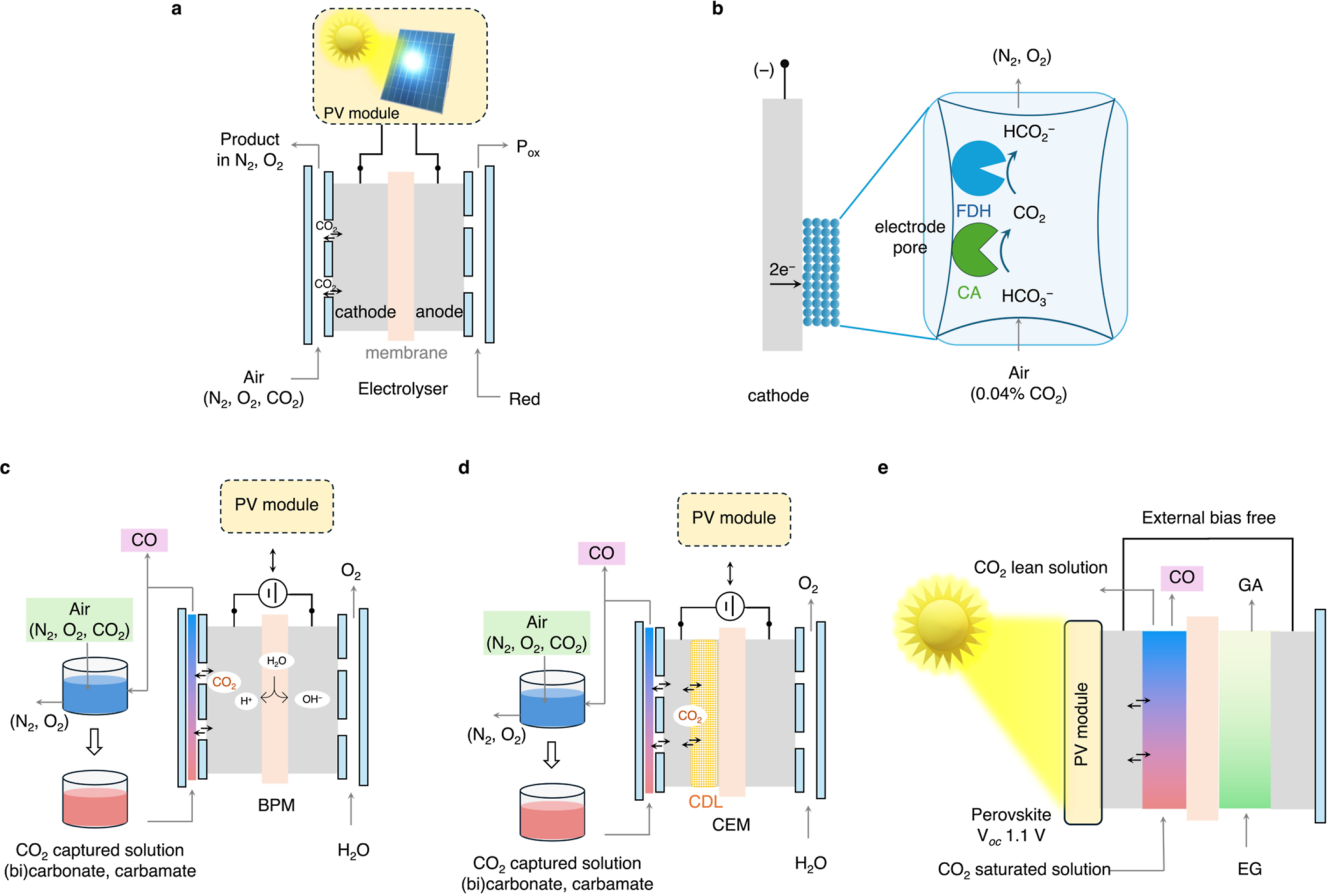
Photovoltaic-driven electrochemical air-to-fuel systems. (a) Photovoltaic (PV)-driven electrolysis directly from air (Red denotes the reductant, *P*_ox_ denotes the oxidized product), (b) carboxysome-inspired semiartificial approach where carbonic anhydrase (CA) converts bicarbonate to CO_2_ for formate dehydrogenase (FDH) inside electrode pores to produce formate from air,^107^ (c) stepwise approach for electrolysis of air-captured CO_2_ solutions using bipolar membranes (BPM) for local CO_2_ release,^74,108^ (d) direct carbonate or carbamate electrolysis with a CO_2_ diffusion layer (CDL) for CO_2_ transport to the cathode^109^ (CEM denotes the cation exchange membrane), and (e) the buried junction photoelectrochemical system operating directly by sunlight under external bias-free conditions^73^ (*V*_OC_, EG, and GA denote the open-circuit voltage, ethylene glycol, and glycolic acid, respectively).

#### 3.2.1 Electrochemical Systems without Photovoltaic Integration

##### 3.2.1.1 Direct Electroreduction from Atmospheric Concentration CO_2_

We have recently reported a nature-inspired approach for CO_2_ capture and electroreduction from atmospheric concentrations, utilizing a local carbon-concentration mechanism involving carboxysome-type microconfined structures, as found naturally in cyanobacteria (Figure 2g). Similar to cyanobacteria, where carbonic anhydrase (CA) and RuBisCO are colocated within a carboxysome, we immobilized carbonic anhydrase and the CO_2_ reductase formate dehydrogenase (FDH) enzymes next to each other within the pores of a mesoporous indium–tin oxide (ITO) electrode (Figure 5b).^107^ During electrolysis in pH ranges 7–8, CA facilitates the local release of gaseous CO_2_ from dissolved bicarbonate, which is then consumed by FDH as a substrate to produce formate. This strategy to maximize the local CO_2_ concentration is particularly promising for low atmospheric CO_2_ concentrations due to the linear Michaelis–Menten-type kinetics observed with enzymes under low substrate conditions, resulting in a 4–8-fold increase in FDH activity for formate production.^110^ A >90% Faradaic efficiency for formate production at −0.6 V vs the standard hydrogen electrode (SHE) was observed, but the current density was low (∼60 μA cm^–2^). Further, the system is oxygen-sensitive due to the observed oxygen reduction reaction under aerobic conditions, a major challenge for direct aerobic CO_2_ reduction.^111^

##### 3.2.1.2 Electroreduction of Chemically Captured Aerobic CO_2_

A promising alternative to direct CO_2_ electroreduction from air involves capturing the aerobic CO_2_ in solution first, followed by its electrolysis (Figure 5c).^72,112,113^ Chemical carbon capture using active chemical agents, such as amines, metal hydroxides, and amino acid salts, is an effective method for storing CO_2_ in solution while separating it from O_2_ and N_2_ (Scheme 1 and Figure 3). The Sargent group reported an electrolyzer system that can directly reduce carbonate electrolyte into synthesis gas, using silver-based catalysts.^74^ This approach used a bipolar membrane-based electrolyzer where membrane-generated local protons during electrolysis migrated to the cathode, resulting in local CO_2_ release from carbonate with subsequent reduction (Figure 5c). Following this strategy, syngas at a 3:1 H_2_/CO ratio was produced by direct carbonate electrolysis without any gaseous CO_2_ contamination, achieving current densities of 150 mA cm^–2^ (energy efficiency ∼30%). However, the required cell potential was high (3.8 V) due to the use of a bipolar membrane and the thermodynamic stability of carbonate salts, and no carbon conversion efficiency (yield of CO produced from CO_2_ supplied) was reported (vide infra for a note on carbon efficiency on captured CO_2_ electrolysis). The cell voltage can be reduced slightly (∼3.3 V at 100 mA cm^–2^) by replacing the bipolar membrane with a cation exchange membrane (CEM) and using a composite CO_2_ diffusion layer (CDL) between the cathode and CEM to facilitate CO_2_ transport from the membrane to the cathode (Figure 5d).^109^

**Scheme 1:**
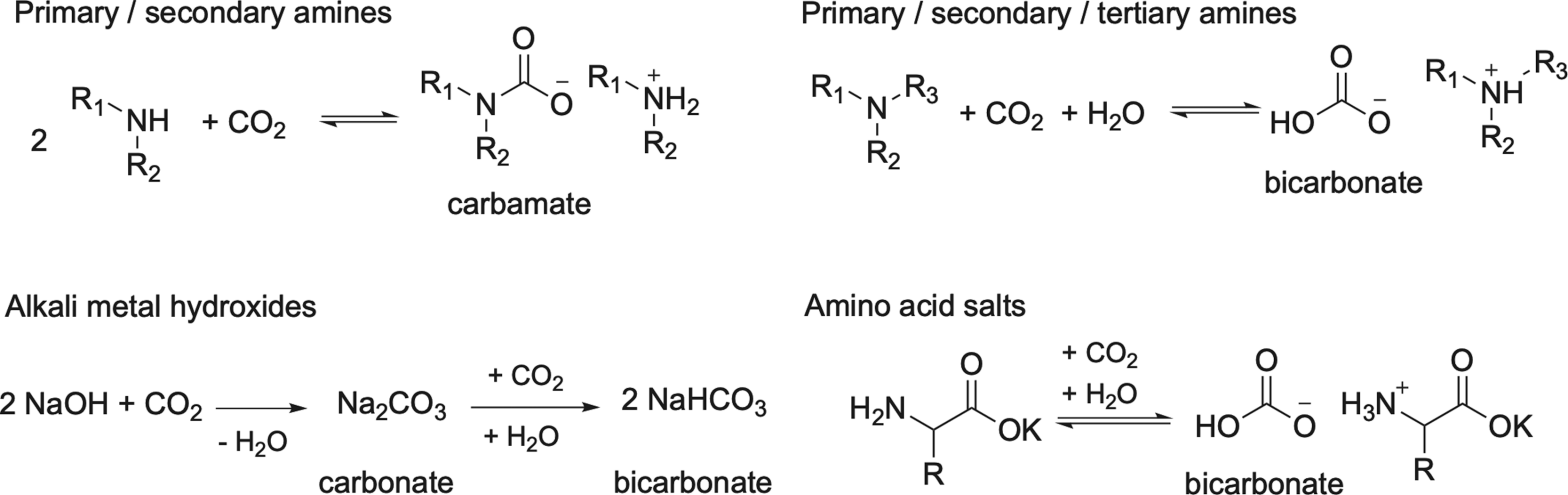
CO_2_ Capture by Different Chemicals with the Chemical Species Formed

Along similar lines, Breugelmans and co-workers reported a bipolar membrane-based zero-gap flow electrolyzer for the electroreduction of direct air-captured (bi)­carbonate solutions into formate or CO using tin oxide or silver nanoparticle-dispersed carbon electrodes (SnO_2_/C or Ag/C, respectively).^114^ Current densities of around 50 mA cm^–2^ were observed at a cell voltage of around 3.5 V, with Faradaic efficiencies for formate or CO production of around 15%. In addition to one-carbon products, multicarbon products such as ethylene can be produced by bipolar membrane-based carbonate electrolysis systems using copper–silver electrodes,^108^ where silver facilitates CO_2_ to CO conversion, and copper induces multicarbon product formation from CO-type intermediates.^115–118^ At a cell voltage of 3.8 V, a carbonate-to-ethylene Faradaic efficiency of 10% was achieved with a carbonate-to-ethylene conversion efficiency of 0.002–0.003%. Besides silver-based catalysts, a copper and cobalt phthalocyanine (CoPc) composite catalyst (Cu/CoPc-CNT, CNT being carbon nanotubes) deposited on carbon paper has also been demonstrated for efficient C2 product formation from carbonate solution electrolysis, with ethylene and ethanol being the main observed products.^119^

Given that alkali (bi)­carbonate is one of the most stable chemical species formed via CO_2_ capture, alternative amino-acid-based electrolytes have also been developed for the capture and direct electrochemical reduction of atmospheric CO_2_.^120^ These systems employ amino acids with basic side chains, such as lysine, arginine, or deprotonated amino acids, such as potassium glycinate (Scheme 1), to capture aerobic CO_2_, followed by the electrochemical reduction of captured CO_2_ using a Zeolitic Imidazolium Framework (ZIF)-8 based nickel single-atom catalyst. Instead of a bipolar membrane, the reported electrolyzer used a simple cation-exchange membrane, producing CO from captured CO_2_ with >50% Faradaic efficiency at a cell voltage of around 3 V and consuming about 30% less energy than alkali hydroxide-based systems.^120^ Apart from the electrochemical, electro-thermochemical hybrid devices are also reported for CO_2_ concentration and conversion to CH_4_ from air, albeit without solar integration.^121^

The carbon conversion efficiency, defined as the fraction of starting CO_2_/(bi)­carbonate molecules converted to reduction products (CO, C_2_H_4,_ etc.), is an important but often overlooked parameter in direct (bi)­carbonate/carbamate electrolysis.^122^ The few studies reporting carbon conversion efficiency indicate that only traces of captured carbon are converted to fuel. The difficulty in carbonate reduction increases with increasing carbon conversion (when the base is regenerated as part of the reaction) as in situ release of gaseous CO_2_ from the remaining carbonate-depleting electrolyte becomes increasingly difficult.^73,108^ Consequently, currents, voltages, and energy and Faradaic efficiencies are expected to falter significantly during the course of the reaction when the carbon conversion efficiency increases, which is of industrial relevance and has not yet been sufficiently addressed. Similar issues persist in photocatalytic CO_2_ reduction, where catalytic activities are typically reported but CO_2_ conversion efficiencies are commonly overlooked (vide infra, Section 3.3).^123,124^

#### 3.2.2 Electrochemical Systems with Demonstrated Photovoltaic Integration

Although the above-mentioned electrolyzers can, in principle, be integrated with photovoltaic-driven renewable electricity generation, this has not been specifically demonstrated. The required cell voltages for these processes (3–4 V) are too high to be efficiently generated by a single light absorber using incident sunlight, necessitating multijunction or multiple solar cells. For example, if integrated with state-of-the-art commercial silicon solar cells (providing open-circuit voltages of 0.6–0.7 V), it will require 4–5 cells connected in series to operate the electrolyzer with incident sunlight, which can make it area-intensive and inefficient. The required cell voltage can be reduced by moving from the thermodynamically demanding water oxidation (*E* ^0^ = −1.23 V) as the counter-anodic reaction to waste-derived alcohol oxidation. The waste oxidation reactions can include the oxidation of glycerol from biodiesel production, ethylene glycol sourced from polyethylene terephthalate (PET) plastic waste, glucose from biomass, or many other waste sources and typically have standard oxidation potentials close to zero (*E* ^0^ ∼ 0 V).^125^

By employing this strategy and utilizing ethylene glycol from PET waste oxidation as the counter reaction, we have shown that aerobic CO_2_ can be captured and converted to syngas using even a single perovskite-based light absorber with an open-circuit voltage of 1.1 V (Figure 5e).^75^ The CO_2_ from the air is captured with a KOH solution dissolved in ethylene glycol and then used directly as an electrolyte in the next conversion step. The solar-driven reduction is performed using a buried-junction photocathode fabricated by integrating a perovskite solar cell with a cobalt phthalocyanine CO_2_-reduction catalyst deposited on multiwalled carbon nanotubes. When immersed in the air-captured CO_2_ solution electrolyte and irradiated with sunlight, the buried junction photocathode produced syngas without any externally applied bias for over 100 h at current densities of around ∼0.1 mA cm^–2^, albeit with a relatively high H_2_/CO ratio (20–25) while selectively oxidizing ethylene glycol to glycolic acid at the anode in a two compartment H-cell separated by a bipolar membrane. For other dilute CO_2_ sources, such as simulated postcombustion flue gas with ∼15% CO_2_ concentration, a higher Faradaic efficiency for CO production (∼20%) and higher current densities (∼1–2 mA cm^–2^) were observed. The carbon conversion efficiency of the process was below 1%.

### 3.3 The Direct Photochemical Pathway

CO_2_ can also be converted into fuels and chemicals using sunlight in a direct photochemical process, without requiring electrode wiring.^126^ In direct photochemical processes, sunlight is captured by a light absorber (molecular or material-based) to generate an electron–hole pair. The electron is then transferred to CO_2_, often facilitated by a cocatalyst, to produce CO_2_-reduction products, while the hole is transferred to a reductant (electron donor) to complete the redox process (Figure 6a).^127^ Adequate bandgap alignment and interfacing between the photosensitizer, catalysts, and substrates are necessary to enable vectorial electron and hole transfer in opposing directions to minimize recombination losses. This is often achieved by bandgap and interface engineering through various methods, including doping, structural modifications, alloying, strain engineering, heterojunction formation, cocatalyst modifications, and others.^128–130^ Simultaneously, the choice of the cocatalyst influences product selectivity, which also, to some degree, depends on the reaction medium. The photocatalytic reduction of CO_2_ using sunlight has been reviewed recently.^126^ Most photocatalytic CO_2_ reduction processes operate under concentrated 100% CO_2_ conditions to ensure high substrate availability without oxygen. Direct photochemical CO_2_ reduction from air is considerably more challenging due to low ambient CO_2_ concentration and oxygen interference.^55,131,132^

**Figure 6:**
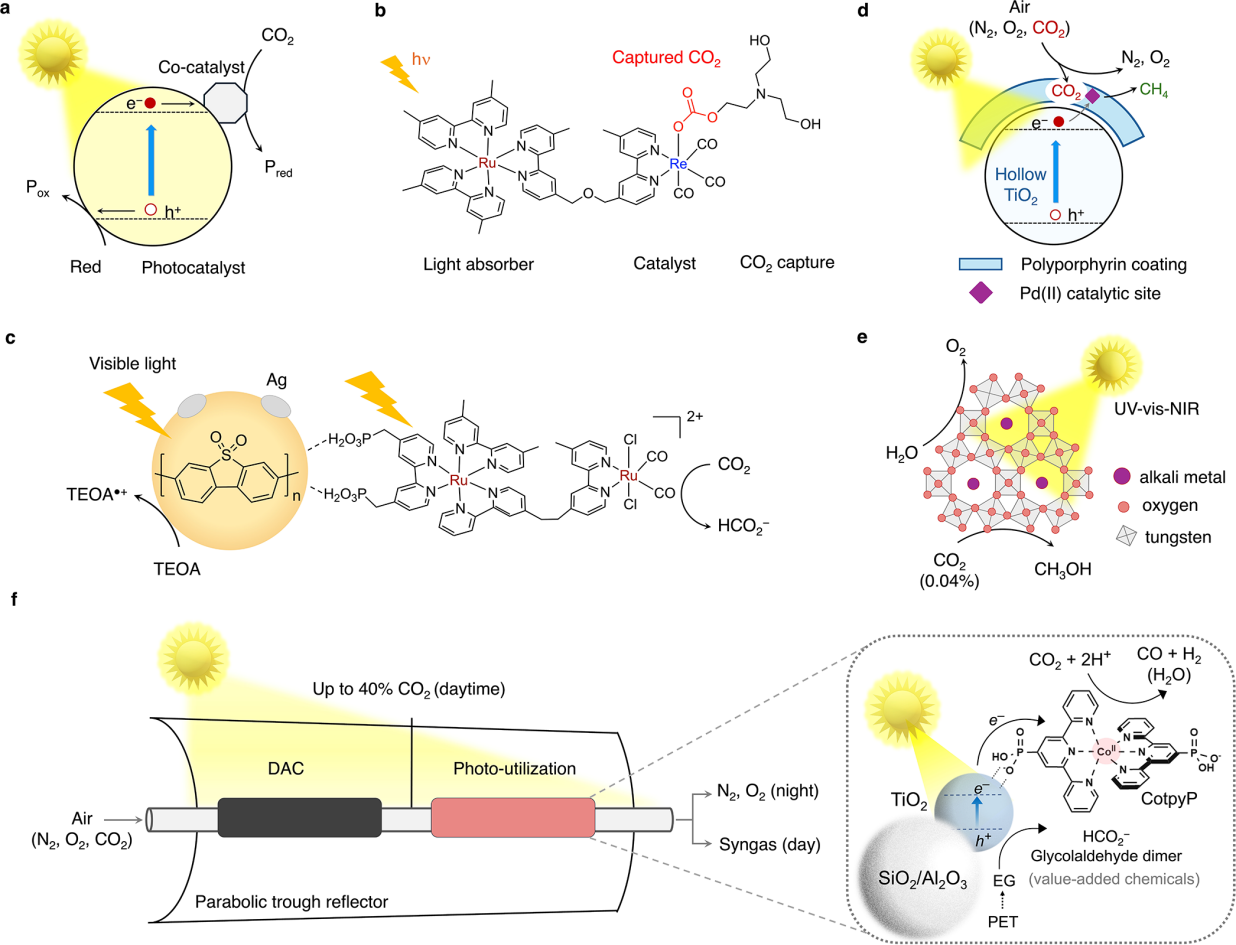
Photochemical solar air-to-fuel technologies. (a) Schematics of photochemical CO_2_ reduction (Red, *P*_red_ and *P*_ox_ denote the reductant, reduction products, and oxidation products, respectively), (b) an example of a bimolecular complex for photochemical CO_2_ conversion from dilute streams,^133^ (c) extended molecular conjugates for photochemical CO_2_ reduction to near quantitative yield using polymer-based visible light absorbers^134^ (TEOA denotes triethanolamine), (d) strategy to enhance local CO_2_ concentration and ward off O_2_ through transparent polyporphyrin coating with embedded Pd­(II) catalytic centers,^135^ (e) CO_2_ reduction from air using face selective hexagonal tungsten bronze materials,^136^ and (f) the dual-bed direct air CO_2_ capture and utilization technology developed by us for solar air-to-fuel synthesis^75^ (DAC: direct air capture, PET: polyethylene terephthalate, EG: ethylene glycol).

#### 3.3.1 CO_2_ Photoreduction from Dilute Concentrations in Anaerobic Concentrations

Ishitani and co-workers (and others) have designed molecular complexes through additive and coordination sphere engineering for CO_2_ photoconversion from dilute streams (including 0.04% CO_2_) under anaerobic conditions via localized CO_2_ capture.^133,137^ In these systems, a light absorber molecule (such as ruthenium tris­(bipyridyl) (Ru­(bpy)_3_)) is typically conjugated with a CO_2_ conversion catalyst (such as rhenium bipyridyl tris­(carbonyl)), while the reaction is carried out with a CO_2_ scavenger in the solution (such as triethanolamine) (Figure 6b).^133^ When light is irradiated onto the solution, the light-absorbing moiety is photoexcited to generate a HOMO–LUMO (electron–hole) pair. Due to suitable alignment of the energy levels (verified in prior experiments), the electron moves to the catalyst, where CO_2_ reduction takes place. Triethanolamine, present in solution, plays a dual role. First, it scavenges CO_2_ from the solution to bring it near the catalytic center via Re–O­(carbonate) bond formation, ensuring local CO_2_ availability even at dilute concentrations. At the same time, triethanolamine also acts as a sacrificial electron donor (reductant), quenching the generated hole at the light absorber while being oxidized. Applying this chemical principle, the reduction of CO_2_ from dilute streams with concentrations as low as 5000 ppm (diluted in argon) has been demonstrated. This corresponds to retaining 60% of the catalytic activity compared to catalysis in pure 100% CO_2_, with a molecular turnover number of 150 after 5 h. A 480 nm wavelength monochromatic light was used, and the system is also active in an electrochemical setup.^138^

The light absorption can be extended to a broader visible region by attaching the molecular structure further to an efficient conjugated polymer-based solar absorber, such as P10, the 10-unit-long homopolymer of dibenzo­[*b,d*]­thiophene sulfone.^139,140^ A recent report by Sprick and co-workers showed near-quantitative CO_2_ conversion to formate under photocatalytic conditions using a similarly designed system at low CO_2_ concentrations (with triethanolamine as the sacrificial electron donor), with a turnover number of around half a million under 460 nm light (Figure 6c).^134^ Catalytic activity under low CO_2_ concentrations is needed to achieve high CO_2_ conversion in any catalytic system but is often overlooked in photocatalytic CO_2_ reduction studies due to low conversion yields typically observed.^141^ Other reports on the developments of low-concentration CO_2_ reduction using molecular systems have been reviewed recently.^137^

It is worth noting that the molecular systems often require sacrificial electron donors (e.g., triethanolamine), which, although useful for catalytic studies to investigate the CO_2_ reduction half-reaction at an early stage, are not practical for large-scale implementation due to their high cost and stoichiometric consumption/decomposition.^142^ Further, molecular systems, due to their well-defined HOMO–LUMO energy gap, often show narrow absorption bands in the solar spectrum (apart from the polymeric systems) that can limit overall efficiencies when using the full solar spectrum. The molecular systems described above are effective for photocatalytic low-concentration CO_2_ reduction only under anaerobic conditions due to their sensitivity to oxygen.

#### 3.3.2 CO_2_ Photoreduction Directly from Air

Direct aerobic CO_2_ photoconversion can be achieved by modifying local catalytic environments with specialized materials to increase the local CO_2_ concentration (for improved rate kinetics and mass transport) and to ward off oxygen, favoring CO_2_ reduction over O_2_ reduction. This was attempted recently by the Wang group, who employed a composite photocatalyst consisting of a hollow TiO_2_ core with surface-coated hyper-cross-linked porphyrin-based polymers coordinated to active Pd­(II) sites (for CO_2_ reduction) to achieve direct aerobic CO_2_ photoconversion to CH_4_ under ultraviolet–visible (UV–vis) light irradiation (Figure 6d).^135^ Here, the TiO_2_ generated electron–hole pairs upon light irradiation and also catalyzed water oxidation as the counter reaction on its surface. The surface-deposited porphyrin-based polymer ensured high CO_2_ availability near the Pd­(II) centers, by capturing CO_2_ from air, with the Pd atoms serving as active catalytic centers for CH_4_ formation. Employing this strategy, the authors reported a 12% conversion yield of CO_2_ to CH_4_ from air after 2 h of UV–vis irradiation (325–780 nm).^135^

Along similar lines, Zhang, Zhao, Sun, and co-workers have reported a series of hexagonal tungsten bronze materials with the formula M_0.33_WO_3_ (M being alkali metals such as K, Rb, Cs) with exposed reactive {010} facets that are active in direct aerobic CO_2_ reduction under UV–vis-NIR illumination (Figure 6e).^136^ These materials were prepared solvothermally from tungsten hexacarbonyl (W­(CO)_6_) and metal nitrate precursors, among which Rb_0.33_WO_3_ showed the most activity in aerobic CO_2_ photoreduction to methanol under full-spectrum radiation (350–2500 nm) or even selective near-infrared (NIR) irradiation (λ > 800 nm), producing methanol with 98% selectivity after 4 reaction hours and ∼4% CO_2_ conversion yield. Doping these structures with molybdenum (3–5%), specifically in Cs_0.33_WO_3_, can further enhance the catalytic activity several-fold (3–4×) under anaerobic conditions.^143^ Other structures, such as rose-like assembled bismuth oxychloride (BiOCl) nanosheets rich in bismuth vacancies with exposed active {001} facets, are also found to be active for CO_2_ capture and photoconversion from air, with activities reaching around 20 μmol g^–1^ h^–1^ for CO production under a UV–vis irradiation for 5 h.^135,144,145^

A few notes on the challenges in aerobic CO_2_ photoconversion and semiconductor photocatalysis in general are warranted here. First, the kinetic activities of these photocatalytic systems remain low due to high charge recombination rates. Consequently, the analyzed product amounts are often small and subject to large errors.^146,147^ In CO_2_ photoreduction specifically, many products, such as CO and CH_4_, can also form from impurity photodegradation.^141^ Therefore, additional verification methods, including robust controls and isotope-labeling experiments, are necessary to identify CO_2_-derived products and calculate true catalytic rates.^148^ This issue is particularly prominent in direct photocatalysis of aerobic CO_2_, where low CO_2_ availability and high oxygen levels further promote the degradation of non-CO_2_ carbon sources such as organics.^75^

#### 3.3.3 CO_2_ Photoreduction Following Direct Air Capture

Besides direct photochemical CO_2_ conversion from air, where the product is typically diluted in O_2_ and N_2_ (Figure 3), CO_2_ can alternatively be precaptured in solution, followed by photoreduction. Han, Liu, and co-workers have shown that CO_2_ from air can be precaptured using a task-specific ionic liquid consisting of a tetrabutylphosphonium cation and a pyridinium 2-oxide anion [P_4444_]­[P-2-O].^144^ Along with atmospheric CO_2_, the ionic liquid also captures atmospheric moisture to form a bicarbonate species. The captured CO_2_ (as bicarbonate) is directly reduced photocatalytically in the next step by using a pyrene-based conjugate polymer. A CO production rate of 47.37 μmol g^–1^ h^–1^ was obtained under visible light irradiation using triethanolamine as the sacrificial electron donor, with around 98% selectivity for CO formation due to suppression of the H_2_ evolution reaction in the ionic liquid medium.

When employing a localized capture (by amines, hydroxides, zeolites) near catalytic centers, it is worth noting that CO_2_ in its captured state is thermodynamically more stable and kinetically more inert than free CO_2_. Thus, while in situ capture increases local CO_2_ concentrations (in the captured form), subsequent conversion is often challenging without prior CO_2_ release.^112^ Addressing this issue, we have designed a stepwise dual-bed direct air-to-fuel reactor that captures, concentrates, and converts CO_2_ from air into CO in a sequential manner using sunlight (Figure 6f).^75^

The photoreactor involves a transparent glass tube reactor with two separate fixed reaction beds—one for DAC and another for CO_2_ photoutilization—mounted on a parabolic trough reflector for sunlight concentration (3–5 kW m^–2^; 3–5 sun) (Figure 6f). The upstream DAC bed contains a solid silica-amine adsorbent to capture CO_2_ from air during nighttime ‘dark’ operation. During the day, solar light is concentrated onto the tube, heating the capture bed to temperatures exceeding 100 °C (a photothermal cover is used for efficient solar heating) and facilitating the release of air-captured CO_2_ in a concentrated stream toward the downstream utilization chamber. Depending on the flow rate, CO_2_ concentrations of more than a thousand times (of air) can be achieved (from 0.04% to >40% v/v). The downstream bed contains molecularly engineered titania nanoparticles with anchored phosphonated Co bis­(terpyridine) CO_2_ reduction catalysts supported on alumina or silica nanopowder (Figure 6f, right) that use concentrated sunlight to convert incident CO_2_ to syngas. PET waste-derived ethylene glycol is presupplied to the conversion bed, which serves as the electron source (reductant), decreases overall energy demand, avoids explosive oxygen-fuel mixture formation, and is transformed into value-added products during the process (all of which may ultimately contribute to the practicality of this process). Although this nascent technology currently exhibits low solar-to-chemical efficiency, future improvements are anticipated, in terms of enhanced catalyst activity, stability, and solar absorption.

### 3.4 The Inorganic–Biological Hybrid Pathway

One primary reason artificial conversion of CO_2_ from air has been challenging is its low ambient concentration, but nature has developed intricate biological mechanisms to address this challenge. Nature’s ability to fix aerobic CO_2_ can be exploited to design a hybrid approach where biological organisms are employed for low-concentration aerobic CO_2_ fixation (via reduction) and integrated with artificial chemical systems to provide the required energy (for example, via solar light harvesting). Among biological organisms, *Cupriavidus necator* (formerly known as *Ralstonia eutropha*) is a soil bacterium with several intriguing capabilities. It can easily adapt between heterotrophic and autotrophic modes, meaning it can grow either by using other organic carbon sources (heterotrophic) or produce its own carbon-containing biomass from inorganic carbon, including atmospheric CO_2_ (autotrophic). In its autotrophic mode, *C. necator* can utilize H_2_ (through hydrogenase enzymes (H_2_ases)) as an energy source to fix ambient CO_2_ into biomass for its growth. Under nutrient-deficient conditions, the biomass formation pathway switches to a carbon-storing mechanism that synthesizes polyhydroxyalkanoate (PHA) at high concentrations instead of forming biomass.^149,150^ Perhaps most intriguingly, the PHA synthesis pathway following CO_2_ fixation under nutrition-deficient conditions can be genetically engineered in this bacterium to produce targeted energy-rich carbon products, including fusel alcohols (C3–C5).^151^

#### 3.4.1 Bioelectrochemistry without Solar Integration

The genetically engineered *C. necator* bacterium’s ability to fix atmospheric CO_2_ into fusel alcohols using H_2_ under nutrient-deficient conditions can be combined with H_2_ production through solar water splitting to synthesize (C3–C5) bioalcohols from atmospheric CO_2_ using sunlight, as demonstrated by Nocera, Silver, and colleagues.^152–156^ Their biohybrid technology, called the Bionic Leaf, merges biological CO_2_ fixation with Nocera’s previously developed artificial leaf technology for solar H_2_ production,^157,158^ enabling solar-powered aerobic CO_2_ conversion into liquid fuels (Figure 7a). A similar approach was previously explored by Liao and colleagues using genetically engineered *C. necator* for electrobiofuel (isobutanol) production, utilizing formic acid as an energy vector.^159^

**Figure 7:**
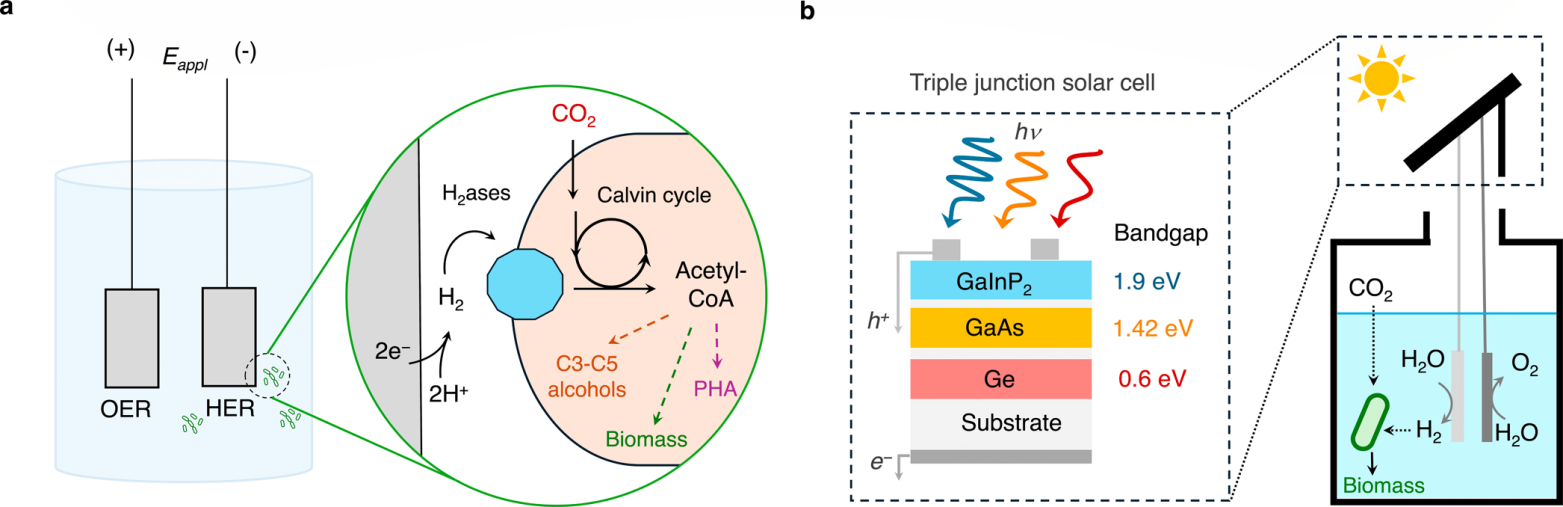
Inorganic–biological hybrid solar air-to-fuel technologies. (a) The electrochemical biohybrid setup for aerobic CO_2_ reduction to C3–C5 alcohols under an electrochemical bias, with a shown biological CO_2_ fixation pathway^152,153^ (OER, HER, and PHA denote the oxygen evolution reaction, hydrogen evolution reaction, and polyhydroxyalkanoate, respectively). (b) The photovoltaic integration of the biohybrid electrolysis setup using an external triple junction solar cell.^154^

Initial reports on the Bionic Leaf focused on the electrochemical aspects, investigating the potential integration of these chemical and biological pathways via intermediate H_2_ production.^153^ Major innovations involved developing efficient water electrolysis systems that can operate under conditions conducive to bacterial growth, including a moderate pH (6–8) and low cell potentials (2–3 V). A self-healing cobalt phosphate (CoP_i_) anode catalyst was found to be effective for catalyzing the oxygen evolution reaction at near-neutral pH, whereas a NiMoZn alloy was used as the cathode catalyst. The electrolyte consisted of CO_2_-saturated phosphate buffer (pH 6.5–7.5; minimal medium) with an added culture of *C. necator* (wild-type or engineered). When this electrochemical cell was operated at a potential of nearly 2.7–3.0 V with wild-type *C. necator*, rapid bacterial biomass growth was observed by optical density analysis due to the consumption of the in situ generated H_2_ by the bacteria. At lower potentials (<2.7 V), a loss in bacterial growth and activity was observed due to the formation of H_2_O_2_ and other reactive oxygen species (ROS). When operated at 3.0 V with a genetically engineered *C. necator* strain in the electrolyte specifically designed for targeted isopropanol synthesis, the selective formation of isopropanol via in situ CO_2_ fixation and reduction was observed (Figure 7a). Isopropanol concentrations in the electrolyte reached 216 mg L^–1^ after 5 days, with 90% carbon selectivity toward isopropanol (current densities around 5 mA cm^–2^, cell voltage 3.0 V).

The required cell potential was later reduced to 2.2 V by using an alternative cathode comprising a cobalt phosphorus (Co–P) catalyst that minimized the formation of H_2_O_2_ and ROS at these low potentials.^152^ Furthermore, the Co–P cathode and CoP_i_ anode can work synergistically to minimize leached cobalt in the electrolyte, enabling it to be transported to and deposited on the anode, leading to an increased cell viability. Using this approach, effective biological fixation of CO_2_ from air (containing 400 ppm of CO_2_) into biomass was demonstrated with wild-type *C. necator* at an overall electrochemical cell voltage of 2.0 V and at current densities of ∼5 mA cm^–2^, with an electricity-to-biomass efficiency of around 20%. Using genetically engineered *C. necator* strains, the product selectivity can be altered to produce C3 or C4 + C5 alcohols, with 25–40% energy efficiencies from pure CO_2_.

#### 3.4.2 Bioelectrochemical with Solar Integration

Low required cell potentials (≤2 V) also enable efficient integration of the biological–inorganic hybrid electrolyzers with solar photovoltaic cells (Figure 7b).^154^ The required cell voltage was obtained from a triple-junction solar cell consisting of Ge, GeAs, and GeInP_2_, with bandgaps of 0.6, 1.42, and 1.9 eV, respectively. The solar cell produced an open-circuit voltage of 2.4 V, with peak power observed near 2.0 V at a current density of ∼10 mA cm^–2^. The attached hybrid electrolyzer contained Co–P and CoP_i_ catalysts as the cathode and anode, respectively, with wild-type *C. necator* in the electrolyte medium for biological CO_2_ fixation using in situ generated H_2_. When no external voltage was applied, the integrated photovoltaic-driven biohybrid electrolyzer operated at a potential difference of approximately 2.3 V across the electrodes with a current density of 7 mA cm^–2^, producing bacterial biomass from pure CO_2_ with an energy (solar-to-chemical) efficiency of around 6.1%. The efficiency was lower than the achievable peak (∼10%, photovoltaic efficiency × electrolyzer efficiency) due to a voltage mismatch between the solar cell output and the electrolyzer input. While this study focused primarily on the utilization of pure CO_2_, activity for CO_2_ fixation from the aerobic atmosphere can be expected based on previous reports. In addition to CO_2_ fixation to biomass or carbon-based alcohols, the bacterium can be engineered to produce fine chemicals, including pharmaceuticals, or even fertilizer (NH_3_) through N_2_ fixation (bionic leaf-N).^160–163^

## 4 Discussion

Several approaches are thus being explored for solar air-to-fuel synthesis to enable future utilization of atmospheric CO_2_. These technologies are at early stages, with technological readiness levels (TRLs) ranging from 2 to 5. Significant technological improvements, both in DAC and in solar-powered CO_2_ conversion, along with their effective integration, are needed to make solar air-to-fuel technologies a market reality. Key features of representative examples of these technological approaches are summarized in Table 1.

**Table 1:** Overview of Representative Solar Air-to-Fuel Technologies

Technological approach	CO_2_ capture method	Conversion technology	Operating temperature ranges	CO_2_ conversions	Products	Solar to fuel efficiency	Major efficiency loss	major advantages	major concerns	tentative TRL^a^	ref
Solar–photothermal	Solid amine-based adsorbents	Thermochemical looping	900–1500 °C	20–40%	H_2_ (75–90%)^b^ CO (6–10%) (further to methanol or kerosene)	1–5%	Radiative heat loss	Mature solar thermal facilities	Area intensity, heat loss, CapEx^f^, critical materials	3–5	^89^
Photovoltaic–electro-chemical	Aqueous hydroxide or amino acid solutions	Direct carbonate and carbamate electrolysis	RT^h^–80 °C	0–10%	H_2_ (50–90%),^c^ and CO (10–50%) or C_2_H_4_ (5–12%) or HCO_2^—^_ (20–80%)	3–8%^e^	Electro-chemical overpotential and resistance PV^g^ efficiency	High areal activity (for electrolysis)	High capex, cell stability, membrane prices, carbon conversion	3–4	^108,109,114,120^
Direct photochemical	Solid adsorbents/photocatalyst material	Solar catalysis using molecules or semiconductors	RT–100 °C	0–10%	H_2_ (20–30%)^b^ and CO (70–80%), or CH_4_ (>90%), or MeOH (>95%)	0.05–1%	Charge carrier recombination Low sunlight absorption	Low CapEx^f^ High scalability	Low activity, material stability	2–3	^75,135,136^
Inorganic–biological hybrid	Biological CO_2_ fixation by *C. necator*	(Photo)electrolysis with microbial CO_2_ conversion	RT–40 °C	90–100%	Liquid fuels (C3 or C4–C5) (55–70%), or biomass (30–45%)	3–6%^d^,^e^	Photovoltaic electrochemical integration PV efficiency	Biological inorganic integration	Scalability, reaction rate	3–4	^152^
											

a TRL denotes the technology readiness level.

b H_2_ generated from coadsorbed moisture.

c H_2_ generated from aqueous solution.

d Assuming a solar cell efficiency of 20%, coupled to the electrolyzer.

e Tentative efficiency for CO_2_ conversion from air, efficiency higher for pure CO_2_.

f CapEx means Capital Expenditure.

g PV denotes photovoltaic.

h RT denotes room temperature.

Currently, the thermochemical pathway (Figure 4) is at tentative TRL values of 3–5. The core technology is being trialed by the spinout company Synhelion to produce solar aviation fuel at several thousand liters per year.^164^ However, the CO_2_ here is captured from concentrated biogenic emissions rather than air to improve economics. The cost of carbon capture increases significantly when transitioning to ultradilute sources such as air. Thus, any fuel produced via photothermal technology following DAC would be more expensive than current biogenic emission-derived fuels. Under optimistic scenarios, the price of synthetic aviation fuel from air-derived CO_2_ produced by this technology is estimated at around $2–$5 per liter, compared to virgin fossil-based aviation fuels at <$1 per liter.^89^ Another recent study has reported a tentative price of air-derived methanol by this route at near $8 per liter, markedly higher than its current market price (<$1 per liter).^165^

The prices can be reduced by increasing the overall solar-to-fuel efficiency of the system from its current 1% to 10%, particularly by optimizing the syngas production process in the solar refinery. This can be addressed by improving heat recovery as radiative heat loss is significant at high operating temperatures (1000–1500 °C) and is being explored as part of the European SUN-to-LIQUID II project.^166^ Opportunities also lie in discovering alternative oxygen-transport materials that can facilitate the thermochemical cycle at lower temperatures,^167,168^ achievable in lower sunlight concentrations and, consequently, reducing radiative heat loss and area intensity (Figure 8). Efforts are ongoing in this regard, focusing on doping ceria with other oxide materials. The use of earth-abundant, noncritical minerals would aid large-scale adoption, and iron-based oxides show promise, but their high-temperature passivation remains a challenge.^169,170^ The mature process of solar thermal electricity generation using concentrated solar power with steam turbines^171^ could help scale up this relatively new fuel generation technology, utilizing existing solar fields. Still, the need for high temperatures, large-area solar fields, high capital expenditure for solar concentrators, large amounts of ceria (a critical mineral), and low specific product yield (mass of product per unit mass of ceria per cycle) could persist as problems for the foreseeable future in the absence of any significant breakthroughs.

**Figure 8:**
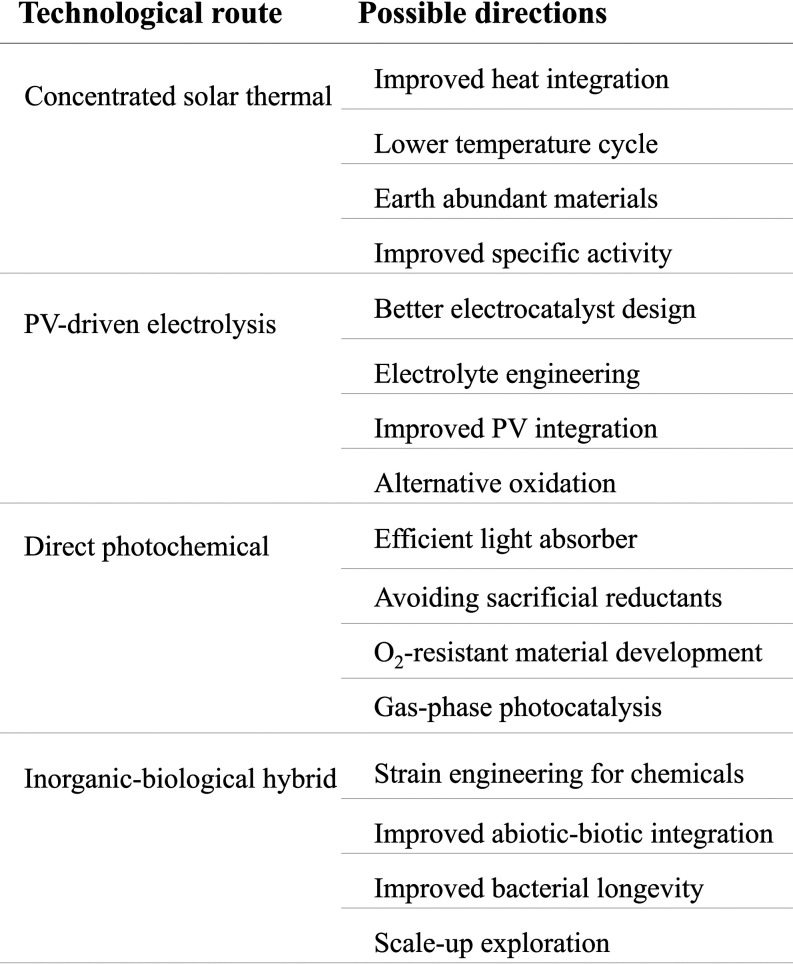
Future research directions among different technological routes.

The photovoltaic-driven electrochemical pathway (Figure 5) does not suffer from significant radiative heat loss (operating at near-ambient temperatures) and is not limited by Carnot inefficiencies. However, the reported developments are still in the early stages. Direct photovoltaic-driven electrochemical reduction of aerobic CO_2_ in solution is more challenging as it combines the difficulty of low CO_2_ concentration with its low solubility in water. Consequently, the initial capture of CO_2_ in an electrolyte solution (using amines, hydroxides, etc), followed by photovoltaic-driven electrolysis, is likely a more manageable approach.^172,173^ The captured-CO_2_ electrochemical reduction systems can achieve high current densities (0.1–0.5 A cm^–2^ levels) and, consequently, high specific area activity. However, the low carbon conversion efficiencies and the high overpotentials required for both the oxygen evolution reaction and the air-captured CO_2_ reduction reaction need to be addressed.^174^ Regarding the latter, electrolyte engineering with different carbon capture solutions could establish a balance between capture and conversion, facilitating rapid direct air CO_2_ capture and its subsequent reduction at low potentials.^112^ Replacing water oxidation with a more energetically favorable waste-derived substrate oxidation can also decrease the overall cell voltage requirement for air-captured CO_2_ reduction (to ∼1 V) while providing a way to valorize waste materials (Figure 8).^175^

New earth-abundant metal-based electrocatalysts can be developed, moving away from noble metals (Au, Ag), which can retain activity in aerobic CO_2_-capturing electrolytes.^176,177^ The long-term stability of captured CO_2_ electrolyzers when operated at high current densities with high carbon conversion efficiencies remains to be explored. The use of bipolar membranes could be avoided when possible due to their high cost, relatively complex fabrication, and high transmembrane electrical resistance at high current densities.^178,179^ Additional operational complexities related to carbonate precipitation and CO_2_ crossover across the membrane must be addressed before the systems can be scaled up. Furthermore, consideration should be given to optimizing the integration of photovoltaic modules with electrolyzers (either as separate units or integrated buried-junction units) to match the photovoltaic module’s output voltage and current to the electrolyzer’s load, to maximize solar-to-chemical efficiencies (Figure 8). Additional complexities arising from low CO_2_ solubility in aqueous electrolyte solutions can be circumvented by moving to gas-phase systems.^180–182^

Direct photochemical air-to-fuel conversion (Figure 6), powered by sunlight, can be developed in a straightforward, scalable setup with low capital expenditure, a key advantage of direct photocatalysis.^183,184^ However, the activities of direct photochemical systems remain too low for their commercial applications. The primary reasons for this are attributed to rapid charge-carrier recombination, incomplete light absorption, and degradation of photocatalyst activity over time.^185,186^ Recombination can be minimized by developing materials containing heterojunctions to ensure facile charge separation upon their photoinduced generation.^187–189^ Similarly, improved light absorption by developing visible-light-responsive photocatalysts needs to be pursued to utilize a broad solar spectrum.^190,191^

During direct photocatalytic aerobic CO_2_ reduction, care must be taken to separate fuels from air after photoconversion to avoid the dangers posed by air–fuel mixtures. Additional difficulty arises from the challenge of competing aerobic O_2_ reduction. The dual-step approach introduced by us provides a workaround by concentrating CO_2_ and removing O_2_ in an upstream gas-phase process.^75^ Still, the efficiencies in both solar CO_2_ concentration and CO_2_ conversion require improvement. The field will benefit from breakthroughs in photocatalyst development,^192^ leading to enhanced light absorption, charge separation, electron transfer, and stability, ultimately bringing it closer to the desirable 5% solar-to-chemical efficiency target (Figure 8).^131,193,194^

The Bionic Leaf technology employs a hybrid approach by utilizing natural systems to facilitate CO_2_ fixation, which is often the most challenging aspect for aerobic CO_2_ conversion, while generating the necessary energy through solar H_2_ production (Figure 7). The bacteria can be genetically engineered to produce a variety of fuels and chemicals, providing access to products that are not readily accessible from purely synthetic devices.^195,196^ Further work can thus engineer bacterial strains for the synthesis of fine chemicals. The use of living organisms, however, requires that contaminants and the medium temperature be controlled during operation to prevent cell death.

Among future explorations, the scalability of this technology for carbonaceous fuel production can be investigated. The current major efficiency loss stems from the efficiency cap of the solar cell. The voltage and current matching between the photovoltaic output and the biohybrid electrolyzer input needs to be tuned to ensure the entire process operates at the maximum possible solar conversion efficiency.^154^ As it currently uses a bacterial batch process for CO_2_ conversion, the reaction rates should be optimized to achieve the highest possible CO_2_ conversion from aerobic CO_2_ in the shortest possible time. Overall, this technological approach holds promise but requires further development beyond its current stage, particularly in terms of scalability and economic viability (Figure 8).

## 5 Outlook

Despite several advances, solar air-to-fuel technologies require substantial further development. The thermochemical route, though the most mature and capable of producing tangible air-to-fuel products, remains capital-intensive and operates at extremely high temperatures that challenge material and system stability. Moving toward lower reaction temperatures using alternative oxygen-transport materials will be crucial. Meanwhile, electrochemical systems, particularly direct carbonate, bicarbonate, and carbamate electrolyzers, show promise but require further research to improve carbon conversion efficiency, prevent crossover and precipitation, enhance stability, optimize energy use, and improve operational flexibility. Technoeconomic analyses under relevant industrial conditions are necessary, with a recent study identifying iridium costs from anodic water oxidation as a significant cost contributor in direct carbonate electrolysis for ethylene production.^197^ Compared to the electrocatalytic air-to-fuel systems, current photocatalytic systems are at a lower TRL (2–3) due to their lower activity. Fundamental research in photocatalyst development for both gaseous and chemically captured CO_2_ photoreduction is required to enhance catalytic efficiencies. Likewise, the biohybrid approach requires further study, particularly regarding system stability, long-term energy efficiency, and technoeconomic factors, before large-scale implementation can be considered.

The world consumes around 15 billion liters of crude oil per day, and substituting this vast amount with sustainable synthetic fuels from air is a massive challenge. Electrification and the hydrogen economy can make significant strides in decarbonizing some industrial sectors, including light transport, but synthetic fuels will be needed to meet the liquid fuel demands of the aviation, heavy transport, and chemical industries to produce plastics as well as bulk and fine chemicals with low carbon or net-zero emissions. The scientific community’s interest in tackling this formidable challenge and making solar air-to-fuel a reality has been evident over the past decade, with new partnerships, consortia, and research centers emerging rapidly.^198^ However, major hurdles in the industrial and political spheres need to be overcome to realize this ambition and to implement commercial clean technologies. Support from government policies for the use of green synthetic fuels, even as a small fraction blended with current fuels, will be necessary for the early adoption of these emerging technologies and place them on the learning curve.^199^ Harvesting and utilizing atmospheric CO_2_ to store solar energy as fuels would enable us to effectively regulate atmospheric CO_2_ levels and meet the energy demands of our civilization while enabling the adoption of a circular carbon economy.^200^ How effectively we can achieve this and how long it will take will have a decisive impact on our society’s future.
